# DaReUS-Loop: a web server to model multiple loops in homology models

**DOI:** 10.1093/nar/gkz403

**Published:** 2019-05-22

**Authors:** Yasaman Karami, Julien Rey, Guillaume Postic, Samuel Murail, Pierre Tufféry, Sjoerd J de Vries

**Affiliations:** 1 Sorbonne Paris Cité, Université Paris Diderot, CNRS UMR 8251, INSERM ERL U1133, Paris, France; 2 Ressource Parisienne en Bioinformatique Structurale (RPBS), Paris, France; 3 Institut Français de Bioinformatique (IFB), UMS 3601-CNRS, Université Paris-Saclay, Orsay, France

## Abstract

Loop regions in protein structures often have crucial roles, and they are much more variable in sequence and structure than other regions. In homology modeling, this leads to larger deviations from the homologous templates, and loop modeling of homology models remains an open problem. To address this issue, we have previously developed the DaReUS-Loop protocol, leading to significant improvement over existing methods. Here, a DaReUS-Loop web server is presented, providing an automated platform for modeling or remodeling loops in the context of homology models. This is the first web server accepting a protein with up to 20 loop regions, and modeling them all in parallel. It also provides a prediction confidence level that corresponds to the expected accuracy of the loops. DaReUS-Loop facilitates the analysis of the results through its interactive graphical interface and is freely available at http://bioserv.rpbs.univ-paris-diderot.fr/services/DaReUS-Loop/.

## INTRODUCTION

Prediction of protein structures is one of the most challenging problems in biology (1). This is reflected by the large number of protein sequences known today (∼109 million) in UniProt versus the number of known protein structures (about 139 thousand) in Protein Data Bank, PDB (2). This means homology modeling is a crucial technique to obtain structural insight (3), and homology modeling methods keep improving significantly (4,5). Loops are regions with often crucial roles in protein-protein interactions, protein function, drug design and docking of small molecules (6–8). Successful loop modeling can lead towards accurate design and engineering of proteins, large peptides, antibodies, drugs or synthetic vaccines, to name a few (9). Despite the development of dedicated loop modeling methods, the overall accuracy of homology models tends to be considerably lower in loop regions, and loop modeling of homology models remains an open problem (10–13).

Loop modeling approaches can be divided into *ab initio* (14–19), data-based (20–24) and the combination of both methods (25–27). *Ab initio* methods explore the conformational space to find loop conformations computationally, while data-based approaches mine a database using the geometry of flanks (a few residues before and after the loop of interest), to search for possible candidates. Many of these methods achieve successful loop predictions in exact environments (i.e. missing loops in crystal structures) (16–18,21,23,24,27). However, few methods have been applied to the prediction of loops in perturbed situations (i.e. homology models) (16,21,23). The difficulty of those cases is reflected by the much lower accuracy of the resulting loop models. Moreover, few methods are available as web servers: principally GalaxyLoopPS2 (16), LoopIng (23), Sphinx (27). In addition to these methods, there are servers for ModLoop (28), ArchPRED (29), FALC-Loop (30) and RCD+ (18), which have only been tested on crystal structures, and there is SuperLooper2 (20), which is an interactive web application rather than an automated web server. Moreover, there are MODELLER (31), Loopy (32), OSCAR-loop (33), Rosetta-NGK (15), LEAP (17) and M-DiSGro (34), available only as tools that have to be installed locally. Finally, there are several web servers that are specific for the prediction of loops in antibodies (27,35–38).

Previously we have proposed DaReUS-Loop, a data-based approach using remote or unrelated structures for loop modeling (39). The method has been validated on benchmarks of loops extracted from CASP11 and CASP12 targets and shown to improve the accuracy of loop modeling, with respect to the state-of-the-art approaches (considering both *ab initio* and data-based methods). In addition, significant improvements have been obtained to predict long loops with at least 15 and at most 30 residues. Importantly, DaReUS-Loop tackles the practical application of loop modeling in non-ideal conditions (homology models) (39).

Here, we describe the DaReUS-Loop web server, an automated platform for modeling or remodeling loops in the context of homology models. The web server uses the same protocol as in the original publication (39), except that in the final minimization, MODELLER (31) (which is not free software) has been replaced by GROMACS (40). For the convenience of the user, the web server accepts a protein with up to 20 loop regions defined, and models them all in parallel. The server assigns a confidence value to every modelled loop, that correlates well with the accuracy of predictions.

## MATERIALS AND METHODS

### Description of the web server

The details of the DaReUS-Loop protocol are explained in (39). The only difference is that final minimisation with MODELLER (31), which is not free software, has been replaced by GROMACS (40) (see Supplementary data for the details). In the current study, MODELLER was still used to generate initial homology models that were subsequently re-modelled using the DaReUS-Loop server.

The minimum loop length is 2, and the maximum loop length is 30 residues. In addition, for the convenience of the user, the web server accepts a protein with up to 20 loop regions defined, and models them in parallel. This has implications on the valid combinations of loop candidates, which is why the server can run in three different modes: remodeling mode, modeling mode, and advanced modeling mode. These options are briefly explained below (see Supplementary data for a more detailed definition).

In all cases, the DaReUS-Loop web server takes as input: (i) atomic coordinates of a protein in PDB format and (ii) a protein sequence in FASTA format.

- **Remodeling:** The server accepts an initial homology model and remodels the loops as indicated by the user in a gapped sequence. In this mode, each loop is being modelled separately, while the other loops are kept in their initial configuration (from the input structure file).
- **Modeling:** The server takes a gapped PDB and completes the missing loops using the full protein sequence provided by the user, in parallel. In addition, the server builds a consensus model, choosing the top candidate of each loop. Then final models for every loop are built using this consensus structure.
- **Advanced modeling:** In this mode, the inputs are similar to modeling mode (a gapped PDB and full protein sequence). Each loop is being modelled independently, while the other loops are omitted as gaps. This mode slightly improves the loop accuracy at the cost of introducing gaps in the final models.

Note that all three modes produce the same results if the input protein has only one loop to be modelled.

It is possible to define a PDB code that will be excluded from the search, for the purpose of benchmarking. In this case, close homologs (those with at least 70% sequence identity) are ignored within the search dataset.

**Figure 1.**
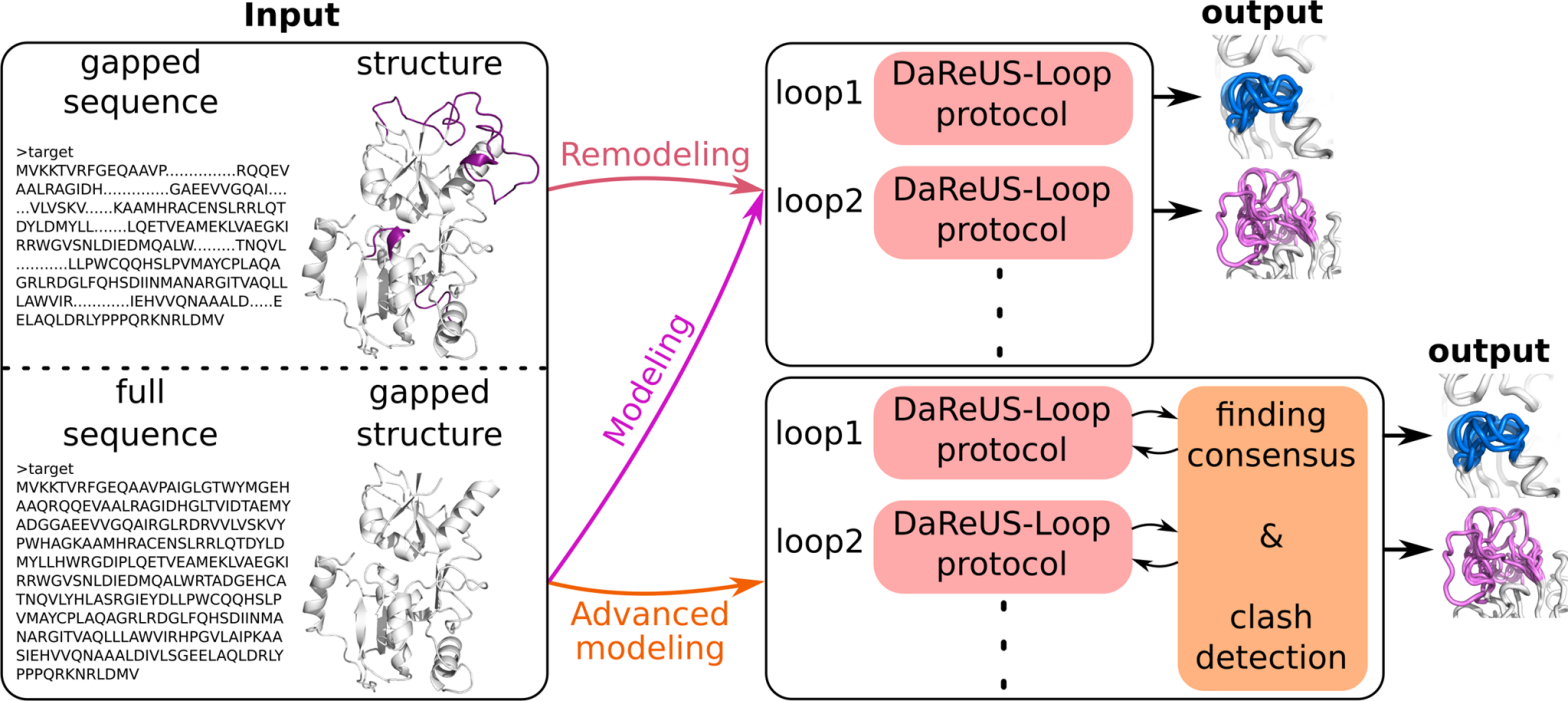
The work flow of DaReUS-Loop web server.

For every loop region, the server returns a maximum of 10 candidate models and a confidence score. In addition, to facilitate the quality assessment of loop candidates, the server returns a table reporting the final GROMACS energy values (40) and another energy measure that is the KORP score (41) for every loop candidate. In case of multiple loops, a general clash report is generated, showing possible clashes between candidates of different loops. The report is useful in the advanced and remodeling modes, since there is a possibility that candidates of one loop have clashes with candidates of other loops. Therefore, the clash report guides the user how to choose different combinations of candidates for each loop to avoid possible clashes. A brief description of the protocol is shown in Figure 1.

The server provides a visualisation facility using the NGL Viewer (42). User can select the modelled loops one at a time and all the final loop candidates will be shown on the structure using different colours. This options facilitates the visual inspection of final models.

All DaReUS-Loop web server results presented in the manuscript are for remodeling mode and all predictions were evaluated using the flank RMSD, as defined in (39).

## RESULTS

### Performance and comparison with other approaches

The DaReUS-Loop web server has been validated on the same test sets as the original DaReUS-Loop protocol, namely the targets of the CASP11 (http://predictioncenter.org/casp11/) and CASP12 (http://predictioncenter.org/casp12/) experiments (43,44). The server results were compared with those of the original DaReUS-Loop protocol and with GalaxyLoop-PS2 (16), Rosetta Next-generation KIC (NGK) (15), RCD+ (18), LoopIng (23) and Sphinx (27).Consequently, the comparisons are grouped by the type of the method: *ab initio* (GalaxyLoop-PS2 and Rosetta) and data-based (LoopIng and Sphinx). However, most of these methods have some limitations, for instance GalaxyLoopPS2 can model loops of maximum 20 amino acids that belong to proteins with <300 residues. In this context, the same subsets as in the original DaReUS-Loop paper were used. Here, set_*ai*_ is the subset of loops where all *ab initio* methods (GalaxyLoopPS2, RCD+, and Rosetta NGK) gave a result. Likewise, set_*db*_ is the subset of loops where both data-based methods (LoopIng and Sphinx) gave a result. This subset was evaluated using 2-residue flanks, since LoopIng does not return more. Each subset was limited to those loops that were classified as high-confidence by DaReUS-Loop. Finally, the loops of the original homology model, as generated by MODELLER (31), were evaluated as reference.

Overall statistics on the best of top 10 models are shown in Table 1. The average performance of the DaReUS-Loop web server is within 0.1 Å of the published DaReUS-Loop protocol. Average performance is better than NGK, GalaxyLoopPS2, RCD+ and MODELLER by at least 0.59, 0.34, 0.80 and 0.94 Å, for the CASP11 and CASP12 test sets, respectively. The remodeling protocol outperforms LoopIng for all sets, with a gain of at least 1.28 Å and outperforms Sphinx by at least 0.89 Å.

**Table 1.** Comparison with state-of-the-art methods

		CASP11	CASP12	<1 Å (%)	<2 Å (%)
set_*ai*_	DaReUS-Loop server	2.00	2.35	20	53
	DaReUS-Loop	**1.91**	**2.30**	**23**	**58**
	NGK	2.59	2.99	15	41
	GalaxyLoopPS2	2.34	2.88	16	45
	RCD+	2.71	3.11	8	41
	MODELLER	2.94	3.52	12	40
	size	40	46		
set_*db*_	DaReUS-Loop server	**2.01**	**2.25**	**19**	**60**
	DaReUS-Loop	2.05	**2.25**	**19**	58
	LoopIng	3.66	3.53	12	23
	Sphinx	2.90	3.19	15	43
	size	51	55		

Average flanked RMSD (Å) are reported for the CASP11 and CASP12 test sets. Comparison is between the DaReUS-Loop web server and the published version, as well as various ab initio methods (Rosetta NGK, GalaxyLoop-PS2, RCD+ and MODELLER) and data-based methods (LoopIng and Sphinx). Since Sphinx is a hybrid method (combination of ab initio and knowledge-based methods), we reported its results along with LoopIng. Results are reported on the common high confidence sub-set of loops that could be predicted by all the methods of the same class (setai and setdb, respectively). All the values reported in this table correspond to the best flanked RMSD (Å) over top 10 models. The percentage of highly accurate predictions (<1 and <2 Å) is also reported. Bold values correspond to the best values among all the methods.

DaReUS-Loop generates high-accuracy loop models (<1 Å) for 20% and medium-accuracy models (<2 Å) for 53% of the cases in the set_*ai*_ subset (Table 1). The results for high accuracy constitute an improvement by 5, 4, 8 and 8% over Rosetta NGK, GalaxyLoop-PS2, RCD+ and MODELLER, respectively. For the set_*db*_ subset, the improvements are of 13% and 10%, respectively, over LoopIng and Sphinx.

A detailed comparison of the methods with respect to different loop sizes is reported in Supplementary Tables S1 and S2, for *ab initio* and data-based methods, respectively.

### The simultaneous modeling and remodeling of multiple loops

The CASP11 and CASP12 benchmarks contain multiple loops per homology model in most of the cases (see Supplementary Figure S1). In the original DaReUS-Loop publication, each loop was re-modelled independently and one at a time. For the server, three different modes were tested: loop remodeling, loop modeling and advanced loop modeling. Detailed results are in Supplementary Table S3. Briefly, it was found that remodeling usually gives the best results, but that advanced modeling is better in some cases. Note that the three modes give the same result if only a single loop is being modelled. The performance is also reported for modeling loops that are connecting different secondary structures. For that, all the loops in the benchmark were divided into three main groups, according to the secondary structures of their flanks: (i) α−α, (ii) α−β and (iii) β−β. The results are shown in Supplementary Table S4), suggesting the performance is the best for modelling loops connecting two different α-helices, and is better for the loops joining one α-helix to a β-strand compared to the loops connecting two different β-strands.

Among the existing tools for loop modeling, Rosetta NGK is the only one that can deal with arbitrary multiple loops. While M-DiSGro is a tool for modeling multiple loops, they must be interacting, i.e. within spatial proximity. Consequently, we are only able to compare the modeling and advanced modeling modes with Rosetta NGK, and with the initial loops from MODELLER (Table 2). In advanced modeling scenario, the average flanked RMSDs for the two test sets are 2.10 and 2.18 Å, respectively. The results are better than Rosetta NGK with average flanked RMSD of 2.61 and 2.63 Å and drastically better than MODELLER (2.97 and 3.15 Å). Also the percentage of high resolution predictions is higher in both modeling modes (16%) compared to Rosetta NGK (14%) and MODELLER (9%).

**Table 2.** Prediction results over the best of top10 models

	CASP11	CASP12	<1 Å (%)	<2 Å (%)
DaReUS-Loop server	**2.10**	**2.18**	**17**	**58**
NGK	2.61	2.63	14	40
MODELLER	2.97	3.15	9	43
size	48	50		

Average flanked RMSD ( Å) are reported for the CASP11 and CASP12 test sets. Comparison is between DaReUS-Loop web server and other methods (Rosetta NGK and MODELLER). All the values reported in this table correspond to the best flanked RMSD ( Å) over top 10 models. The percentage of highly accurate predictions (<1 and <2 Å) is also reported. Bold values correspond to the best values among all the methods.

To the best of our knowledge, among all existing loop modeling web servers, only the GalaxyLoop server accepts multiple loops at the same time. Still, the maximum number of loops is limited to three, and in the original publication (39), GalaxyLoop was only validated on single-loop test cases. This makes DaReUS-Loop the first web server to be validated on the simultaneous modeling of multiple loop regions in homology models.

### Results on a concrete example

**Figure 2.**
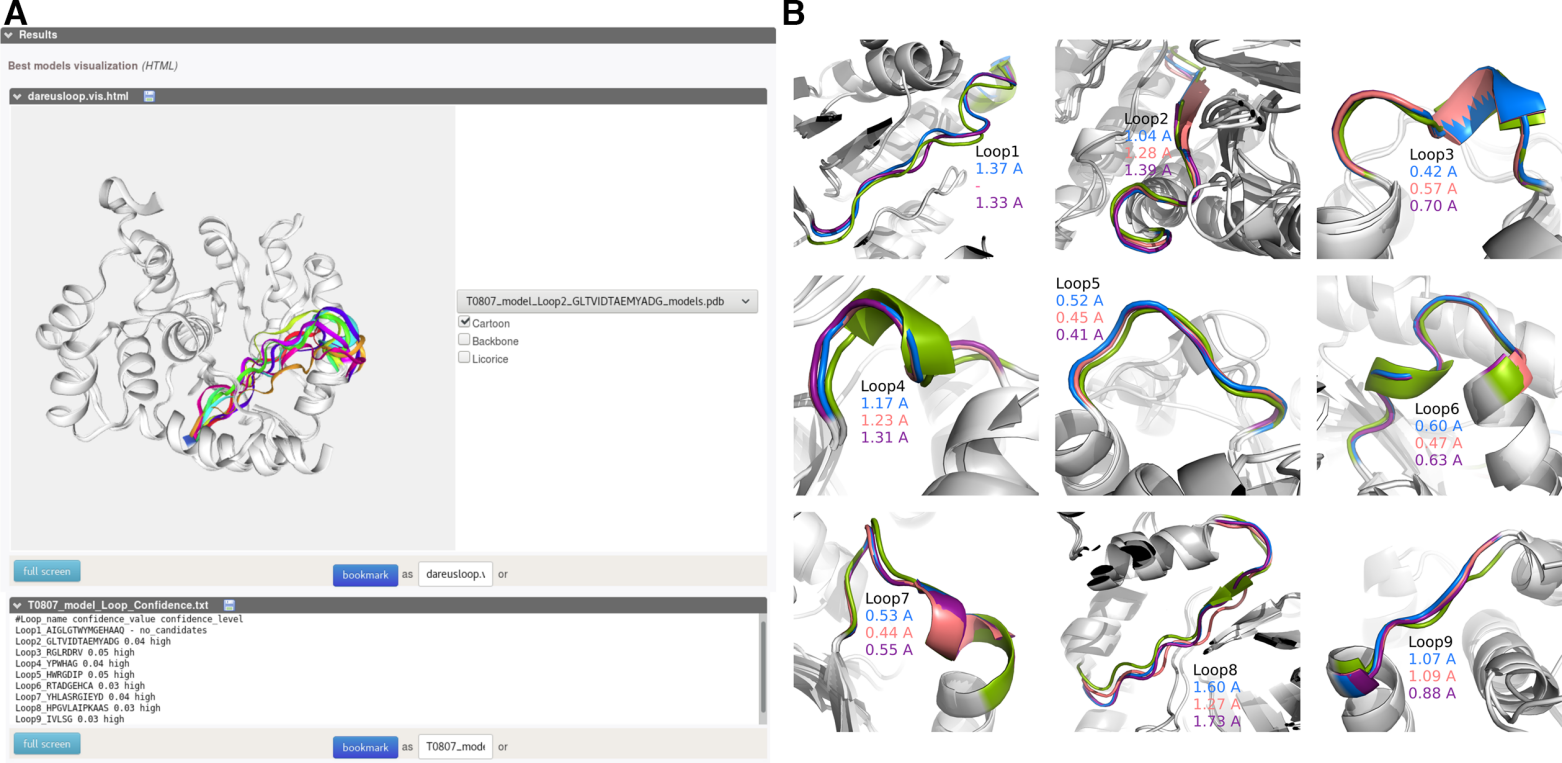
Illustrative example of DaReUS-Loop performance. Nine loops from the homology model of T0807 in CASP11 test set are modelled separately, in each of the three modes (remodeling, modeling and advanced modeling). (**A**) Example of result page provided by the web server for modeling loop number 2 (residues 45–58), using the modeling scenario. Top: final top 10 candidates predicted by the server are visualised on the structure using different colors. Bottom: the confidence values and levels for every loop are reported. (**B**) For every loop, the best predictions of each model are shown (remodeling: blue, modeling: pink and advanced modeling: purple) and the native loop conformations are depicted in green. The loops are spanning the following residues, respectively: 16–30, 45–58, 69–75, 82–87, 113–119, 154–162, 168–178, 234–245 and 258–262. The RMSD of the top candidate is reported with respect to the native loop conformation, after fitting on the flanks.

One of the CASP11 targets, T0807 is selected as an illustrative example to demonstrate the performance of our web server. From the homology model, nine loop regions are identified: 16–30, 45–58, 69–75, 82–87, 113–119, 154–162, 168–178, 234–245 and 258–262. First, the server was run in remodeling mode, providing the initial homology model and a gapped sequence to re-model the loops. In addition, the gapped model along with full sequence was given to the web server selecting first “modeling mode” and then ‘advanced modeling mode’. Final predictions for loop 2 (45–59) in modeling mode and the confidence report for all the loops are shown in Figure 2A). Next we measured the backbone RMSD between the best loop candidate (out of top 10) and the native loop conformation (PDB id: 4WGH), after superimposing the flanks. The results for all the modes and the native conformation are reported on Figure 2B) for every loop.

### Computational time

The performance of DaReUS-Loop is not dependent on the protein size, but it depends on the number of loops to be modeled, as well as their sizes. The computational time needed for modeling a single loop might be within the range of 20–30 min, whereas for a protein with 10 loops or more the runtime may vary between 40 and 120 min, depending on the loop sizes. On average, the running time is between 40 and 50 min, however it highly depends on the traffic load of the cluster.

## CONCLUSION

The DaReUS-loop web server relies on a data-based approach for loop modeling. Compared to previous web servers, it comes with two main advancements that are (i) improved modeling of loops in homology models, and (ii) a demonstrated ability to model several loops simultaneously. Our results show that for >50% of the loops in the test benchmark, loops can be modeled with <2 Å RMSD from the native loop conformations, taking the lowest value among the 10 predicted loop candidates.

An interesting perspective for future research is the combination of DaReUS-Loop with template-based docking methods (45–47). Template based docking provides a prediction of an entire protein-protein complex as a low-resolution model that needs to be refined (48). This is complementary to loop (re)modeling, the high-resolution refinement of loops in a single protein. This must be balanced with other forms of refinement, in particular that of the relative orientations of the protein chain.

We stress that DaReUS-Loop is a consensus method that does not rank the 10 predicted candidates. In case of multiple loops, the Cartesian combination of all loop candidates must be considered. Therefore, one direction for future research is an investigation into effective scoring function able to identify the best combination of candidates among those arising from multiple loops. However, the prediction of multiple candidates could also be considered as an advantage. For example, a flexibility score could be conceived that reflects the predicted degree of conformational flexibility/diversity for every loop. We expect it could provide a starting point to assess the impact of loop conformation on the generation of models for protein-protein complexes. In future research, knowledge about disulfide bridges will be integrated into the protocol. Finally, DaReUS-Loop could be integrated with experimental data or with residue contact prediction, which could be used as effective filters of combinations of predicted loop candidates.

## DATA AVAILABILITY

The set of all models for CASP11 and CASP12 generated and analysed during the current study, as well as the results obtained from the web server in all the three modes (remodeling, modeling and advance modeling) are provided as a dataset (Harvard Dataverse, https://doi.org/10.7910/DVN/ZI6ROT). The results from other methods were reported in the previous manuscript (39).

## Supplementary Material

gkz403_Supplemental_Files
