# Supplementary material for: DaReUS-Loop: a web server to model multiple loops in homology models
Source: Nucleic Acids Res. 2019 May 22;47(W1):W423–8. doi: 10.1093/nar/gkz403 (PMC6602439; doi:10.1093/nar/gkz403)
Supplement: gkz403_Supplemental_Files [file gkz403_supplemental_files.pdf]

Supporting data: DaReUS-Loop: a web server to model multiple loops in homology models

Yasaman Karami[1,2], Julien Rey[1,2], Guillaume Postic[1,2,3], Samuel Murail[1], Pierre Tufféry[1,2,*], and Sjoerd J. de Vries[1,2,*]

[1]Sorbonne Paris Cité, Université Paris Diderot, CNRS UMR 8251, INSERM ERL U1133, Paris, France.
[2]Ressource Parisienne en Bioinformatique Structurale (RPBS), Paris, France.
[3]Institut Français de Bioinformatique (IFB), UMS 3601-CNRS, Université Paris-Saclay, Orsay, France
[*]pierre.tuffery@univ-paris-diderot.fr, sjoerd.de-vries@inserm.fr

Energy minimisation

Final energy minimisation were conducted using Gromacs 2018 [1], the CHARMM36m force field [2] and the steepest descent algorithm for 1000 steps. All bonds were constrained using the LINCS algorithm. The particle mesh Ewald algorithm was used to handle electrostatics with a $10\mathring{A}$ cutoff for the short-range part and a grid spacing of $1.2\mathring{A}$ for the long-range contribution in reciprocal space. The Verlet buffer scheme was used for non-bonded interactions, the neighbor list was updated every 20 steps.

Loop size dependent performance

Detailed comparisons of the server with other *ab initio* and data-based methods are reported in **Table S1** and **Table S2**, respectively to represent the performance for different loop sizes. The comparisons are performed over the common high confidence sub-set of loops that can be modelled using all the methods.

Simultaneous modeling of multiple loops

The original DaReUS-Loop protocol is based on modeling a single loop at a time: it requires an initial model and a single loop region, which is re-modeled.

In contrast, the web server can accept homology models with multiple loop regions, up to 20. As shown in the flowchart (see **Figure 1**), it is still based on considering a single loop region at a time, but it runs in parallel, predicting one loop while keeping the other loops constant. The server supports three different approaches on how to treat these other loops, in order to avoid clashes among them. Note that this is irrelevant if there is only one loop region.

- Remodeling: The other loops are kept in their initial configuration (from the input structure file).
- Modeling: The server first builds a consensus model, choosing the top candidate of each loop. Note that other candidates might be considered in case of loop-loop clashes between top candidates. Then final models for every loop are built using this consensus structure.
- Advanced modeling: All loops are modeled independently, and all other loops are omitted as gaps. In this mode the loop accuracy is slightly improved at the cost of introducing gaps in the final models.

Table S1: **Loop dependent comparison with the *ab initio* methods.** Average flanked RMSD (Å) are reported for different loop sizes of the CASP11 and CASP12 test sets. Comparison is between the DaReUS-Loop web server, as well as Rosetta NGK, GalaxyLoop-PS2, MODELLER and RCD$^+$. Results are reported on the common high confidence sub-set of loops that could be predicted by all the methods of the same class. All the values reported in this table correspond to the best flanked RMSD (Å) over top 10 models. The percentage of high accurate predictions, $< 1.0Å$ and $< 2.0Å$, has been reported in paranthesis for each method and loop size. Bold values correspond to the best values among all the methods.

size	#loops	DaReUS-Loop server	NGk	GalaxyLoopPS2	MODELLER	RCD+
5	17	1.74 (18, 65)	**1.38 (35, 76)**	**1.38 (47, 76)**	1.49 (24, 76)	1.62 (12, 65)
6	13	**1.48 (38, 77)**	1.90 (31, 62)	1.92 (23, 54)	2.26 (23, 46)	1.93 (31, 69)
7	13	**1.62 (31, 62)**	2.59 (15, 38)	2.11 (15, 54)	3.12 (8, 38)	2.45 (8, 46)
8	8	**1.93 (25, 50)**	2.39 (0, 25)	2.36 (0, 38)	2.66 (0, 25)	2.41 (0, 38)
9	7	**3.00 (14, 43)**	4.70 (0, 0)	4.40 (0, 14)	5.32 (0, 0)	4.62 (0, 14)
10	9	2.93 (11, 11)	2.89 (0, 33)	**2.71 (0, 33)**	4.02 (0, 33)	3.24 (0, 22)
11	4	**2.24 (25, 50)**	3.80 (0, 0)	2.50 (25, 25)	2.86 (25, 25)	4.98 (0, 0)
12	4	**2.93 (0, 25)**	3.03 (0, 25)	3.74 (0, 25)	4.60 (0, 50)	3.12 (0, 25)
13	1	1.86 (0, 100)	**1.58 (0, 100)**	2.91 (0, 0)	3.96 (0, 0)	3.41 (0, 0)
14	5	**2.51 (0, 60)**	3.85 (0, 20)	3.36 (0, 40)	4.40 (20, 20)	3.76 (0, 40)
15	1	**1.37 (0, 100)**	3.05 (0, 0)	2.87 (0, 0)	3.22 (0, 0)	2.50 (0, 0)
16	2	4.21 (0, 50)	**2.55 (50, 50)**	3.53 (0, 50)	8.17 (0, 0)	2.92 (0, 0)
17	2	**5.12 (0, 0)**	12.52 (0, 0)	10.64 (0, 0)	6.92 (0, 50)	11.70 (0, 0)

Table S2: **Loop dependent comparison with the data-based methods.** Average flanked RMSD (Å) are reported for different loop sizes of the CASP11 and CASP12 test sets. Comparison is between the DaReUS-Loop web server, as well as LoopIng and Sphinx. Results are reported on the common high confidence sub-set of loops that could be predicted by all the methods of the same class. All the values reported in this table correspond to the best flanked RMSD (Å) over top 10 models. The percentage of high accurate predictions, $< 1.0Å$ and $< 2.0Å$, has been reported in paranthesis for each method and loop size. Bold values correspond to the best values among all the methods.

size	#loops	DaReUS-Loop server	LoopIng	Sphinx
5	22	**1.70 (27, 73)**	2.06 (23, 55)	1.75 (32, 73)
6	15	**1.60 (33, 80)**	1.90 (33, 47)	2.31 (13, 60)
7	16	**1.62 (25, 75)**	2.83 (6, 12)	2.53 (19, 50)
8	11	**1.84 (18, 55)**	3.59 (0, 0)	3.05 (0, 18)
9	5	**2.36 (20, 60)**	5.25 (0, 0)	4.35 (0, 0)
10	10	**3.04 (0, 10)**	4.09 (0, 0)	3.55 (0, 30)
11	4	**2.23 (25, 50)**	4.54 (0, 0)	3.70 (25, 25)
12	6	**2.79 (0, 33)**	4.42 (17, 17)	4.43 (0, 33)
13	2	**1.62 (0, 50)**	4.38 (50, 50)	3.27 (50, 50)
14	5	**2.27 (0, 80)**	5.16 (0, 20)	3.60 (20, 40)
15	2	3.95 (50, 50)	8.22 (0, 0)	**3.30 (50, 50)**
16	3	4.61 (0, 33)	6.84 (0, 0)	**3.94 (0, 33)**
17	4	**3.26 (0, 50)**	7.82 (0, 0)	8.72 (0, 0)
22	1	**1.37 (0, 100)**	7.62 (0, 0)	4.48 (0, 0)

All three modes were tested using the two CASP11 and CASP12 test sets. The average backbone flanked RMSD between the best of top 10 predictions and the native targets are shown in **Table S3**. Note that results are reported for the high confidence predictions. In summary, we found remodeling mode to be (on average) the most accurate, followed by advanced modeling mode, followed by modeling mode. Starting from the initial homology models, remodeling mode performs slightly better than our previous single loop

	**CASP11**	**CASP12**	$< 1\AA$ (%)	$< 2\AA$ (%)
Remodeling	2.10	**2.18**	**17**	**58**
Modeling	2.49	2.57	16	53
Advanced modeling	**2.09**	2.26	16	51
size	48	50		

Table S3: **Prediction results over the best of top10 models.** Average flanked RMSD ($\AA$) are reported for the CASP11 and CASP12 test sets. Comparison is between the three prediction modes of the web server. All the values reported in this table correspond to the best flanked RMSD ($\AA$) over top 10 models. The percentage of highly accurate predictions ($< 1\AA$ and $< 2\AA$) is also reported. Bold values correspond to the best values among all the methods.

	$\beta - \beta$	$\alpha - \alpha$	$\beta - \alpha$
Remodeling	2.44	1.64	2.05
Modeling	3.11	1.78	2.31
Advanced modeling	2.42	1.75	2.12
size	40	23	35

Table S4: **Prediction results over grouped by the secondary structures.** Average flanked RMSD ($\AA$) are reported for the CASP11 and CASP12 test sets. Loops are divided in to the following three groups according to the secondary structures flanking them: *(i)* $\beta - \beta$, *(ii)* $\alpha - \alpha$ and *(iii)* $\beta - \alpha$. Comparison is between the three prediction modes of the web server. All the values reported in this table correspond to the best flanked RMSD ($\AA$) over top 10 models.

modeling method, DaReUS-Loop. On the other hand, starting from gapped homology models, advanced modeling performs better than modeling scenario and on par with the two remodeling and single loop modes.

We speculate that the reason why remodeling works the best is that the initial model often contains decent (albeit suboptimal) conformations for most of the loops. If the user has expert knowledge on which loops are initially poor, we believe that advanced loop modeling works better. Another use case for advanced loop modeling would be if experimental information are available. In that case, after advanced loop modeling, the Cartesian combination of all loop candidates could be considered, applying the experimental data as a highly selected filter.

We report the performance of the server for modeling loops that connect different secondary structures in **Table S4**. For that, all the loops in the benchmark have been divided into three main gropus, according to the secondary structures of their flanks: *(i)* $\alpha - \alpha$, *(i)* $\alpha - \beta$ and *(i)* $\beta - \beta$. The results of each modeling modes have been reported for every group. Results suggest that the performance is the best for modelling loops connecting two different α-helices, and is better for the loops joining one α-helix to a β-strand compared to the loops connecting two different β-strands.

Limitations of the web server

Initial homology model DaReUS-Loop requires an initial homology model to (re)model the loops. The model must already have the correct sequence, *i.e.* raw template structures are not accepted. The residue numbering must start from 1. DaReUS-Loop can model the loops of a single protein chain, that may contain only standard amino acids. DaReUS-Loop will model missing side chain atoms using OSCAR-star [3], but all backbone atoms must be present.

Protein sequence: The protein sequence may contain only standard amino acids. and must follow exactly the same numbering scheme as of the structure. For example, if the protein has residue "ALA 16", the 16th letter of the sequence (including gaps) must be "A".

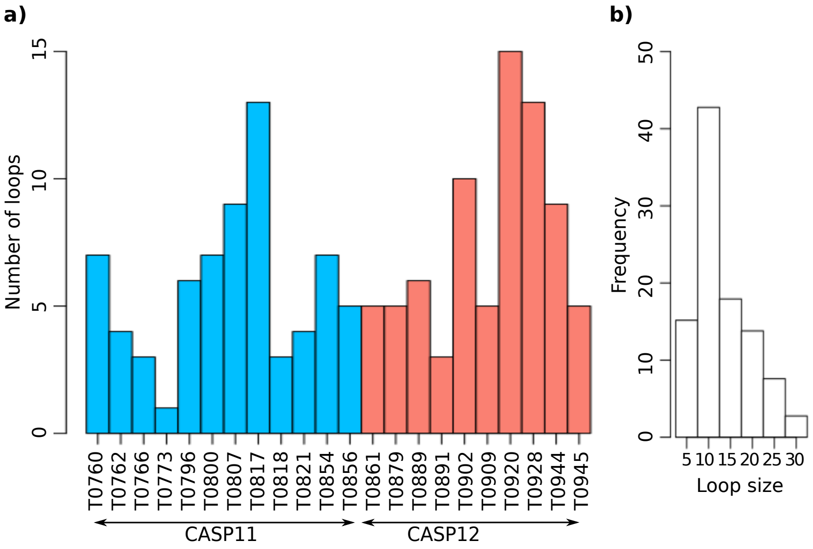

Figure S1: Distribution of loops in the test set. **(a)** Number of loops for every CASP target is reported. **(b)** The size of the loops is within the range of 5-30 amino acids and their frequencies are depicted here.

Loop length: Loops are represented as gaps in the input sequence or structure. Between two gaps, at least four non-gap residues must be present. The minimum loop length is 2, and the maximum loop length is 30. DaReUS-Loop cannot model N- or C-termini. It is still recommended to provide full sequences rather than truncated ones, since this leads to better sequence profiles. Otherwise, missing N- or C-termini in sequence or structure are ignored. The maximum number of loops in the structure is 20.

Initial model quality: DaReUS-Loop assumes that, other than the (gapped) loop regions, the initial homology model is of decent quality (TM-score > 0.5). In particular, the flank regions (the four residues adjacent to each gap) must be accurate. It is highly recommended to define gaps such that all flanks are in a helix or sheet region of the homology model.

References

[1] Szilárd Pall, Mark James Abraham, Carsten Kutzner, Berk Hess, and Erik Lindahl. Tackling exascale software challenges in molecular dynamics simulations with gromacs. In *International Conference on Exascale Applications and Software*, pages 3–27. Springer, 2014.

[2] J. Huang, S. Rauscher, G. Nawrocki, T. Ran, M. Feig, B. L. de Groot, H. Grubmuller, and A. D. MacKerell. CHARMM36m: an improved force field for folded and intrinsically disordered proteins. *Nat. Methods*, 14(1):71–73, 01 2017. [PubMed:27819658] [PubMed Central:PMC5199616] [doi:10.1038/nmeth.4067].

[3] S. Liang, D. Zheng, C. Zhang, and D. M. Standley. Fast and accurate prediction of protein side-chain conformations. *Bioinformatics*, 27(20):2913–2914, Oct 2011. [PubMed:21873640] [PubMed Central:PMC3187653] [doi:10.1093/bioinformatics/btr482].
